# Small molecules promote the in vitro expansion and passaging of adult mouse pancreatic islets

**DOI:** 10.1016/j.bbrep.2026.102622

**Published:** 2026-05-20

**Authors:** Lingxian Zhang, Kairui Liu, Xiaoxiao Zhang, Yubing Cui, Hao Hu, Xiaolei Hu

**Affiliations:** a Department of Endocrinology, The First Affiliated Hospital of Bengbu Medical University, Bengbu, 233000, China; b Department of Health Management, Jiangxi Provincial People's Hospital, The First Affiliated Hospital of Nanchang Medical College, Nanchang, 330000, China; c Institute of Organoid Technology, bioGenous Biotechnology Inc, Suzhou, 215125, China

**Keywords:** Islet-like organs, Culture medium, Expanded

## Abstract

Islet-like organs can provide a new solution for the treatment of diabetes and personalized drug testing. However, because adult pancreatic tissue lacks regenerative capacity, it is still not possible to expand islet clusters from adult mice in vitro or to further passage them. Here, we describe a small molecule-based culture medium that contains specific compounds that can amplify pancreatic clusters from adult mice; these clusters also express markers similar to those of primary pancreatic islets. In addition, the short passage of pancreatic islet clusters can be achieved through their aggregation on microporous plates. Further mechanistic analysis revealed that WS6+GABA in the culture medium contributes to the expansion of pancreatic islets, while AS8351 contributes to the expression of insulin in pancreatic islet clusters. Finally, we developed a simple mouse islet eyeball transplantation device, which increases the simplicity and speed of islet transplantation. We constructed an expandable functional islet-like organ culture system derived from adult mouse pancreatic tissue and expanded the adult tissue source of islet-like organs, which are important steps for the in vitro expansion of islet tissue and the development of culture media. It is crucial for the future treatment of diabetes, especially for regeneration of the pancreas, and the use of personalized drugs that a large number of islets be derived from the same person.

## Highlights

• Constructed an expandable functional islet-like organ culture medium that can enlarge the islet clusters of adult wild-type mice.
• WS6+GABA contributes to the expansion while AS8351 contributes to the expression of insulin in the culture medium.
• Developed a simpler mouse eyeball transplantation device.

## 1 Introduction

Diabetes is a chronic metabolic disease that affects human health [1]. The number of patients with diabetes continues to increase, which has imposed a substantial economic burden on society. However, traditional drug treatment measures cannot resolve the issue of insufficient insulin secretion from the pancreas. Researchers are also exploring other methods, such as islet transplantation [2], but islet donors are limited. The field of organoid research has rapidly developed in recent years. Organoids are derived from stem cells and are three-dimensional cell cultures that feature some key characteristics of organs. Using various technologies, researchers have successfully cultivated various organoids from human or mouse tissues and organs, including the small intestine [3], brain [4], liver [5], kidney [6], and pancreas [7]. To meet the clinical and research needs, numerous strategies have been established for the construction of artificial islets as well as islet organoids [8].The emergence of pancreatic organoids has led to the expectation that β-like cells within these organoids will infinitely expand and secrete insulin in vitro.

Due to the insufficient regenerative ability of the adult pancreas, most pancreatic islet-like organs originate from iPSCs or hPSCs. For example, in 2009, based on the developmental pattern of the pancreas, Zhang et al. gradually induced the expression of pancreas-specific transcription factors, and consequently, cells derived from induced pluripotent stem cells (iPSCs) were successfully differentiated into insulin-producing cells [9]. Felicia W. Pagliuca et al. utilized human pluripotent stem cells (hPSCs) and systematically tested the effects of various factors, including the concentrations and exposure times, on pancreatic precursor cells with regard to their ability to generate glucose-responsive cells that produce the monohormone insulin [10]. A few adult-derived pancreatic islet-like organs, such as Procr + cells, were screened and isolated in 2020 and cocultured with fresh endothelial cells in vitro to obtain functional pancreatic islet-like organs [11]. However, Procr + cells account for only 1% of pancreatic islet cells, and the yield after sorting is very low. In addition, a large number of pancreatic islet cells and endothelial cells are required for each passage. In addition to screening the stem cells of the pancreatic islets themselves, adult nonpancreatic cells, such as those in the liver [12], bile ducts [13], and gastrointestinal tract [14], can also be used to obtain insulin-secreting cells through transdifferentiation. However, this method involves genetic technology, and practical operations are cumbersome and complex.

Currently, only a few reports on how to amplify complete pancreatic islet clusters in vitro have been published. In a 2020 study, a complete small molecule culture medium was developed that can amplify pancreatic islet clusters isolated from pregnant mice and wild-type rats in vitro [15]. However, the amplification of wild-type mouse pancreatic islet clusters, which are more commonly used in biological experiments, has not been reported. Here, we propose a new culture medium formulation that can amplify cell clusters isolated from wild-type mice in vitro. The pancreatic islet clusters cultured in this medium can not only expand but they also exhibit sustained insulin expression. Further mechanistic analysis suggested that the combination of WS6 [16] and GABA [17] in the formula is crucial for the expansion of pancreatic clusters, while AS8351 [18] is crucial for insulin expression in these clusters. In the passage of pancreatic islet-like organs, we found through microplate aggregation that after aggregation, the primary and P0 generation pancreatic islets can continue to expand, which resulted in the transient passage of pancreatic islet-like organs in whole form.

Finally, to increase the simplicity and speed of pancreatic islet transplantation, we chose the anterior chamber of the mouse eye as the transplantation site. According to existing reports, the anterior chamber can serve as a window for real-time observation of the graft in vivo and can be used to determine the state of the graft without harming the host [19]. Therefore, we developed a simple mouse islet eyeball transplantation device based on existing technology [20]. This device is not only inexpensive and readily obtainable but is also easy to assemble and easy to use for transplantation. We identified a small molecule culture medium that can amplify adult mouse pancreatic islet clusters in vitro and induce the clusters to express insulin, achieving transient passage of adult pancreatic islets. The islet eyeball transplantation device we developed will increase the convenience and efficiency of pancreatic islet transplantation.

## 2 Results

### 2.1 Whole small molecule culture medium for in vitro expansion of pancreatic islet clusters

**Fig. 1:**
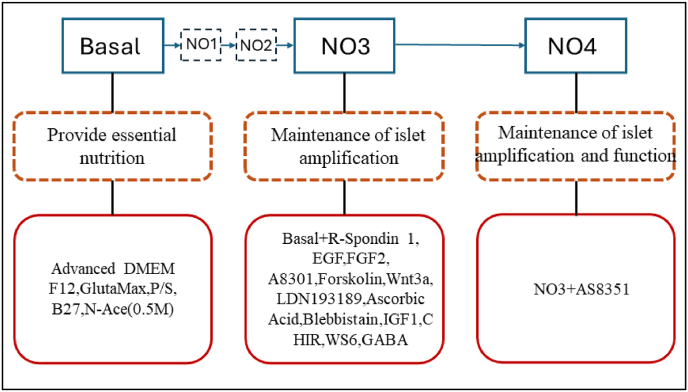
Process for establishing culture media to maintain pancreatic islet expansion and functionality Through a literature review and screening of small molecules, the creation of this culture medium required a total of three stages, namely, the basal basic culture medium stage, the expandable NO3 culture medium stage, and the functional NO4 culture medium stage. The small molecules required for each stage are shown in the figure.

A 2020 study reported that a culture medium formula called PIEM was able to amplify pancreatic islet clusters isolated from pregnant and wild-type rats in vitro, but the medium could not amplify pancreatic islet clusters from wild-type mice, and our study has seamlessly filled this gap. After the process of establishing and improving basic medium (basal), we proceeded to establish NO1 medium (Supplementary Table 1), NO2 medium (Supplementary Table 2), amplification medium (NO3) and functional medium (NO4). Finally, we established a total small molecule culture medium composed of 14 chemicals and hormones, as well as the general nutrients nicotinamide, B27, and glutamine. This culture medium strongly supports the in vitro amplification of pancreatic islet clusters isolated from wild-type mice as well as insulin expression. We named this culture medium NO4. As shown in Fig. 1, we screened the following small molecules in basal media through a literature review and experimental analysis and added them to the medium to create an NO3 amplification medium. On this basis, we continued to add the small molecule AS8351 and ultimately determined the best composition of the NO4 functional culture medium. Table 1 shows the composition, concentration, and literature sources of the chemical compounds in the culture medium.

**Table 1:** Composition of the culture medium for pancreatic islet expansion and functional maintenance.

Ingredient	Volume or Concentration	References
Basal culture medium		
Advanced DMEM F12	50 ml	
GlutaMax	500 μl	
B27	1 ml	
N-Ace(0.5 M)	100 μl	
P/S	200 μl	
Chemicals or hormones		
R-Spondin-1	500 ng/ml	D DOI:10.1016/j.jdsr.2019.03.001 [21]
EGF	50 ng/ml	DOI:10.1038/s41598-021-90643-3 [22]
FGF2	10 ng/ml	DOI:10.1186/s12967-019-1799-1 [23]
A83-01	50 nM	DOI: 10.1186/s12967-019-1799-1 [24]
Forskolin	10 μM	DOI: 10.1183/13993003.00508-2021 [25]
Wnt3a	50 ng/ml	DOI: 10.3390/genes11080939 [26]
LDN193189	100 nM	DOI: 10.1038/s41596-021-00560-y [27]
Ascorbic Acid	0.5 mM	DOI: 10.1263/jbb.105.586 [28]
Blebbistatin	10 μM	DOI: 10.1021/acs.jmedchem.8b00503 [29]
IGF-1	100 ng/ml	DOI: 10.1002/hep.510290632 [30]
CHIR	3 μM	DOI: 10.1038/nature18624 [31]
WS6	1 μM	DOI: 10.1021/ja309304m [16]
GABA	100 μM	DOI: 10.1054/tice.2002.0217 [17]
AS8351	3 μM	DOI: 10.1016/j.stem.2022.01.010 [18]

**Note:**Table 1 shows the required compounds for the culture medium, as well as the required volumes or concentrations and references for each compound.

### 2.2 In vitro amplification of primary pancreatic islet clusters

**Fig. 2:**
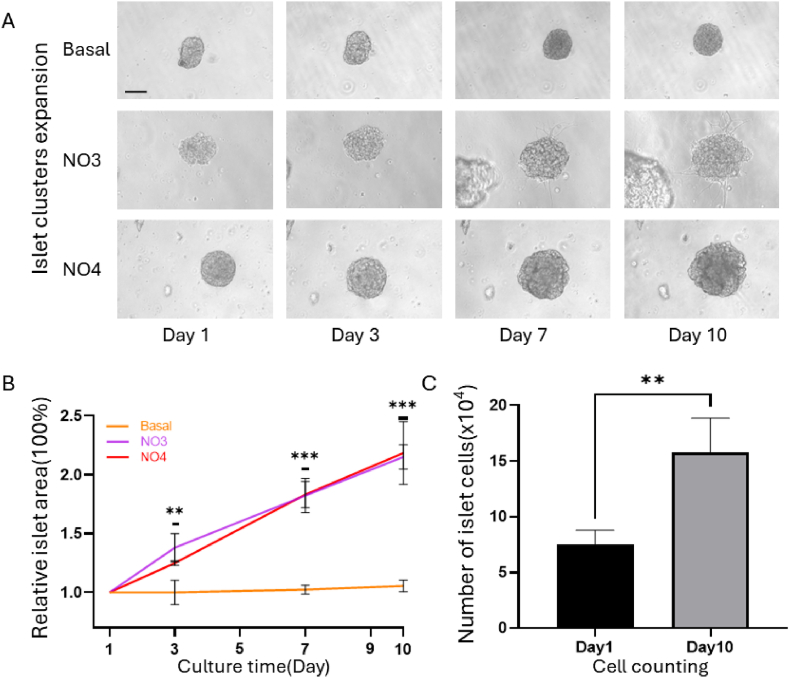
In vitro amplification of pancreatic islet clusters A: Growth field diagram of organoids generated from C57BL/6 mice. The three groups of pancreatic islets were cultured in basal culture medium, NO3 culture medium, or NO4 culture medium. Changes in surface area of cells on days 1, 3, 7, and 10 were measured; scale bar = 100 μm; B: Curves showing the change in surface area of pancreatic islet-like organs in the 3 groups, * *P < 0.01***P < 0.001; C: Comparison of pancreatic islet cell count between the overnight pancreatic islet group on day 1 and the NO4 pancreatic islet group on day 10, with statistical data expressed as the mean ± standard deviation, * *P < 0.01.

To verify the ability of our culture medium to amplify pancreatic clusters from wild-type mice in vitro, C57BL/6 mice were selected for the experiment, and pancreatic islets were isolated and purified (100-200 islets were isolated from each adult mouse). The obtained islets were randomly divided into three groups, embedded in matrix gel, and cultured in 48-well plates. Basal culture medium, NO3 culture medium, and NO4 culture medium were added at a constant temperature. After 1, 3, 7, and 10 days of cultivation, an inverted microscope was used to obtain images at 10x magnification. As shown in Fig. 2-A, in the bright field diagram of pancreatic islet-like organs, the surface area of the pancreatic islet-like organs in the basal group did not significantly change from day 1 to day 10, while the surface area of the pancreatic islet-like organs in the NO3 and NO4 groups significantly expanded. As shown in Fig. 2-B, the surface area of the organoids grown in basal culture medium did not increase within 10 days, while the surface area of the organoids in the NO3 and NO4 groups increased 2.0-2.5 times from day 1 to day 10.

Simultaneously, after the isolation and purification of pancreatic islets, 20 ± islets were randomly divided into two groups for overnight culture and culture in NO4 medium. Then, the islets in the overnight (OVN) group cultured for 1 day (as the initial number of islet-like organs) and those in the NO4 group cultured for 10 days were digested into single cells and counted. As shown in Fig. 2-C, the average number of pancreatic islets in the overnight pancreatic islet group cultured for one day was 7.5 × 10^4^/ml. On the 10th day, the average number of pancreatic islet cells in the NO4 group was 15.75 × 10^4^/ml, almost twice the number of cells on day 1. In summary, basal culture media cannot amplify pancreatic islet clusters isolated from wild-type mice in vitro, while our NO3 and NO4 culture media can increase the surface area of pancreatic islet clusters by approximately twofold in 10 days.

### 2.3 Expanded pancreatic islet clusters maintain insulin secretion

**Fig. 3:**
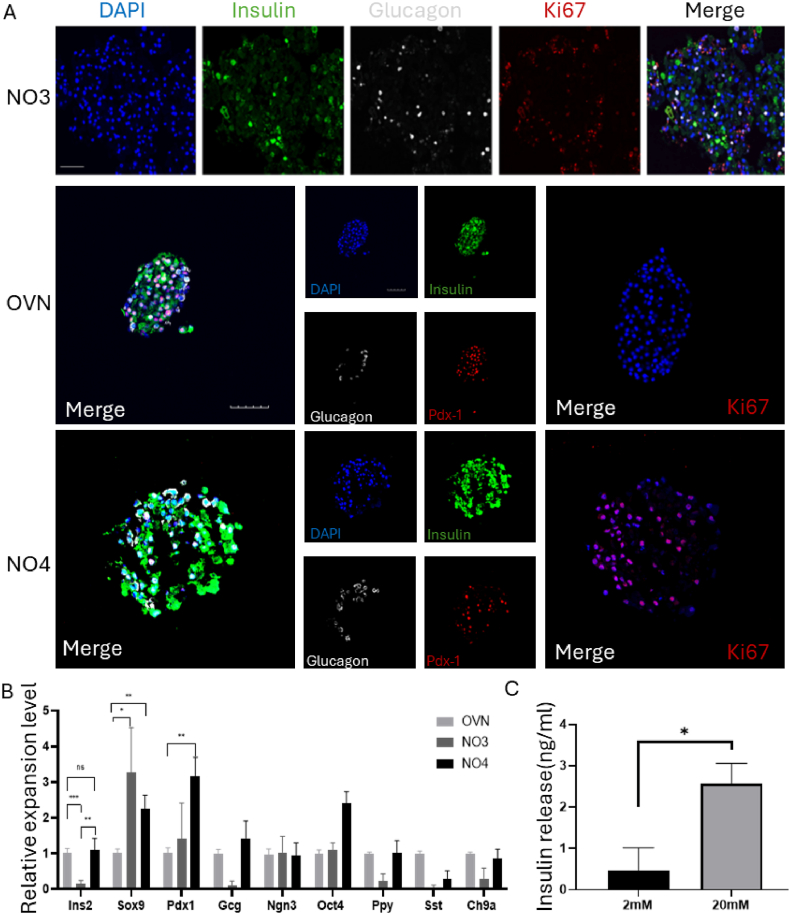
Expanded pancreatic islet clusters maintain insulin secretion A: Immunofluorescence staining of pancreatic islets cultured overnight in NO3 and NO4 media: NO3 group islets, blue: DAPI, green: insulin, white: glucagon, red: Ki67; OVN group and NO4 group islet-like organs, blue: DAPI, green: insulin, white: glucagon, red: Pdx1/red: Ki67, scale bar = 50 μm B: overnight islets, qRT‒PCR experiments on islet-like organs revealed no significant difference between the NO3 and NO4 groups, ns, *P < 0.05, * *P < 0.01, * * *P < 0.001; C: The glucose stimulation experiment of NO4 pancreatic islet-like organs, in which approximately 30 pancreatic islet-like organs were stimulated with 2 mM and 20 mM glucose, *P < 0.05.

To verify that the pancreatic islet-like organs in this culture medium still maintain the cellular characteristics of primary pancreatic islet clusters, immunofluorescence staining was performed to detect insulin (Ins) and glucagon, as these two hormones correspond to the two main hormone-secreting cells of the pancreas, β cells and α cells, which work together to maintain the stability of blood sugar in the body. Immunofluorescence staining was performed on the islet-like organs of the OVN group, NO3 group, and NO4 group. The images were obtained under an inverted fluorescence microscope at 40X magnification, as shown in Fig. 3-A. Both the islet-like organs of the NO3 and NO4 groups expressed insulin and glucagon, which indicates that islet-like organs can continue to secrete insulin while they expand. Moreover, immunofluorescence staining was performed to detect Pdx1 to determine the specificity of the pancreatic islet cells and to further confirm the pancreatic characteristics of the organoids. Ki67 staining represents the expansion ability of cells, and a clear comparison can be seen in the figure. The expansion ability of pancreatic islets in the NO3 and NO4 groups was greater than that of pancreatic islets in the overnight group.

Next, we used qRT‒PCR to detect the expression of key pancreatic cell markers, including Ins2, Sox9, Pdx1, Gcg, Ngn3, Oct4, Ppy, Sst, and Ch9a, in the expanded pancreatic islet cell population. As mentioned earlier, Ins2, Gcg, Ppy, and Sst are markers of insulin, glucagon, pancreatic peptide, and somatostatin, respectively, while Sox9, Pdx1, and Ngn3 are markers of pancreatic islet cells, and Ch9a is a marker of endocrine cells. As shown in Fig. 3-B, in addition to the expression of various pancreatic biomarkers, the expression of Ins2 in the islet-like organs of the NO4 group was significantly greater than that in the NO3 group and approached the expression level observed in pancreatic islets in the overnight group. This indicates that organoids retain the ability to secrete insulin while they expand.

To further verify the functional integrity of the expanded pancreatic islet-like organs, we performed a glucose-stimulated insulin secretion experiment. First, the pancreatic islet-like organs were subjected to 2 mM glucose stimulation for 30 min to collect the supernatant and then they were subjected to 20 mM glucose stimulation for 30 min after which the supernatant was collected again. Two samples were tested for insulin secretion using an ELISA kit. As shown in Fig. 3-C, the organs in the NO4 group secreted an average of 0.47 ng/ml insulin under low-glucose stimulation and an average of 2.56 ng/ml insulin under high-glucose stimulation, which indicates that the pancreatic islet organs cultured in the NO4 medium secrete insulin.

In summary, based on these three experiments, we determined that pancreatic islet-like organs cultured in NO4 medium can not only expand in vitro but can also maintain insulin secretion.

### 2.4 The important roles of WS6, GABA, and AS8351 in the culture media

**Fig. 4:**
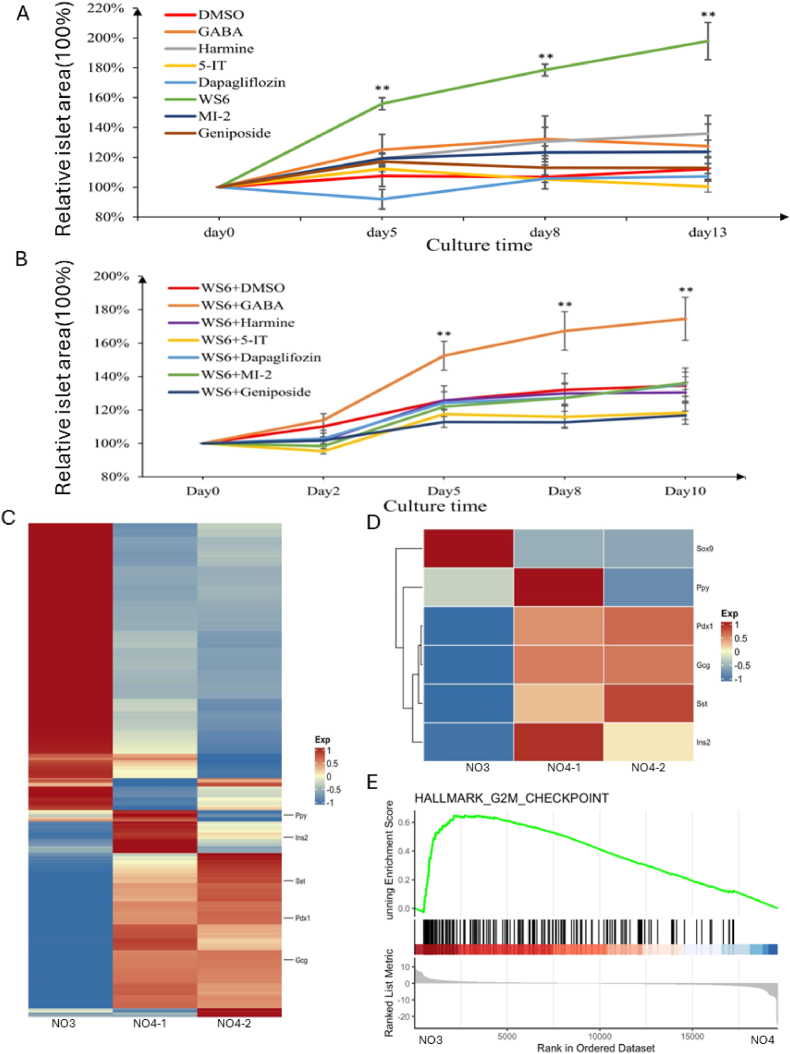
**The Important Role of the Small Molecules WS6, GABA, and AS8351 in Culture Media** A: Growth curve of organoids after the addition of a single small molecule to NO1 medium for screening. Compared with the other groups, the WS6 group was significantly different * *P < 0.01. B: After adding WS6 to medium 1, other small molecules were added separately for combination screening, * *P < 0.01. C: RNA-seq whole-genome heatmap. From left to right, the first column represents NO3-type organs, the second column represents NO4-1-type organs, the third column represents NO4-2-type organs, and each row represents a characteristic gene. The colors from blue to red indicate the relative gene expression levels from low to high; D: The selection of genes that represent pancreatic islet specificity for heatmap analysis shows that from left to right, the first column represents NO3-1 class organs, the second column represents NO4-1 class organs, and the third column represents NO4-2 class organs, with each row representing a characteristic gene. The colors from blue to red indicate the relative gene expression levels from low to high; E: The GSEA diagram of the NO3 and NO4 groups shows NO3-enriched genes on the left and NO4-enriched genes on the right.

We discovered the small molecule WS6 during the development of NO3 medium, and when we screened the small molecules, GABA was found to exert a significant effect on the expansion of pancreatic islet clusters. We observed a significant increase in the surface area of the organoids on day 5 when they were cultured in NO1 media during the first stage (Fig. S1–A). The qRT‒PCR results showed that the expression of the precursor cell marker genes Pdx1, Ngn3, and Sox9 in the organoids cultured for two weeks was greater than that in pancreatic islets cultured overnight, while the expression of Ins2 was relatively lower (Fig. S1–B). Immunofluorescence showed that the organoids expressed insulin but that Ki67 was expressed at lower levels (Fig. S1–C). We then sought to further improve the amplification ability of the organoids, as shown in Fig. 4-A. After analyzing the existing research on promoting pancreatic islet clusters, a literature search on cell growth and replication revealed the following seven small molecules: GABA, Harmine, 5-IT, Dapagliflozin, WS6, MI-2, and Geniposide. Based on the recommended concentrations found in the literature, the compounds were added to the NO1 medium to form 7 different media as determined by the research group, while DMSO was used as the control to cultivate pancreatic islets. Images were obtained under a microscope on days 0, 5, 8, and 13. Multiple repeated experiments revealed that the proliferation rate of the culture medium supplemented with the small molecule WS6 ranged from 150% to 200%, which was significantly greater than that of the other groups. According to recent studies, another 5 small molecules were combined with WS6 and added to NO1 medium to form 6 new combination media. The islets in each group were tracked and observed on days 0, 2, 5, 8, and 10. As shown in Fig. 4-B, the proliferation rate of islets cultured in the WS6+GABA combination medium was significantly greater than that of islets cultured in the other combination media. After multiple experiments, a consistent conclusion was reached. Our data indicate that the pancreatic islet cells in the experimental group had a significantly increased growth rate. Therefore, we named the new combination culture medium Mouse Pancreatic Organ Amplification Medium 2 (NO2). In this study, the small molecule combination of WS6+GABA successfully and greatly enhanced the expansion ability of pancreatic islet-like organs. Next, we drew inspiration from the culture medium used to induce the differentiation of hPSCs into endocrine cells and added CHIR99021, LDN193189, VC, and WNT3A to NO2 medium. We also added a new small molecule, blebbistatin, and the growth factor IGF-1, which are beneficial for organoid growth, and named this combination Mouse Islet Organ Amplification No. 3 Culture Medium (NO3). Islets cultured in NO2 medium served as the control group, and according to the above screening method, the proliferation rate was assessed on days 0, 2, 5, 8, and 13. NO3 medium had a greater amplification advantage than NO2 medium, and the proliferation rate obtained from repeated experiments exceeded 200% (Fig. S1–D).

4-Aminobutyric acid (GABA) is synthesized from glutamate via glutamate decarboxylase. It is generated not only in the nervous system but also in the pancreas, where it is formed within β cells. When ATP levels in the cytoplasm continue to rise or are high in sugar, the generation of GABA and its transformation upon release from β cells are inhibited. In addition, GABA can also promote insulin secretion and reduce blood sugar concentrations, thereby improving the symptoms of diabetes and reducing complications. The diarylurea compound WS6 is considered a novel small molecule that can stimulate pancreatic islet cell growth in rodents and humans. When the glucose concentration increases, WS6 can cause an increase in adenylate cyclase activity. Erb3 binding protein-1 can bind to histone deacetylase and retinoblastoma protein and can inhibit cell cycle regulatory genes. IκB kinase is involved in regulating NF-κB. As part of the proinflammatory stress response of the NF-κB pathway, WS6 can promote the synergistic effect of these two proteins on β-cell proliferation.

Next, we collected RNA from organoids obtained from NO3 medium and organoids obtained from NO4 medium for genomic analysis, as shown in Fig. 4-C.

We found significant differences in the expression distribution of genes related to pancreatic islet-like organs between the NO3 and NO4 groups. Fig. 4-D shows that the NO4 group of organoids expressed more pancreatic islet markers than did the NO3 group of organoids. The GSEA plot in Fig. 4-E further confirmed that the NO3 group of organoids expressed more genes related to cell expansion, while the NO4 group of organoids expressed more genes related to cell maturation. A literature review revealed that AS8351 is a type of KDM. The inhibition of KDM5B by AS8351, a 5B inhibitor, may be crucial for reconstructing the oocyte/2c-specific H3K4me3 domain, thereby promoting the transition from pluripotency to multipotency [18]. However, further exploration is needed to determine whether this site is associated with promoting pancreatic islet maturation.

### 2.5 The limited passage of pancreatic islet clusters

**Fig. 5:**
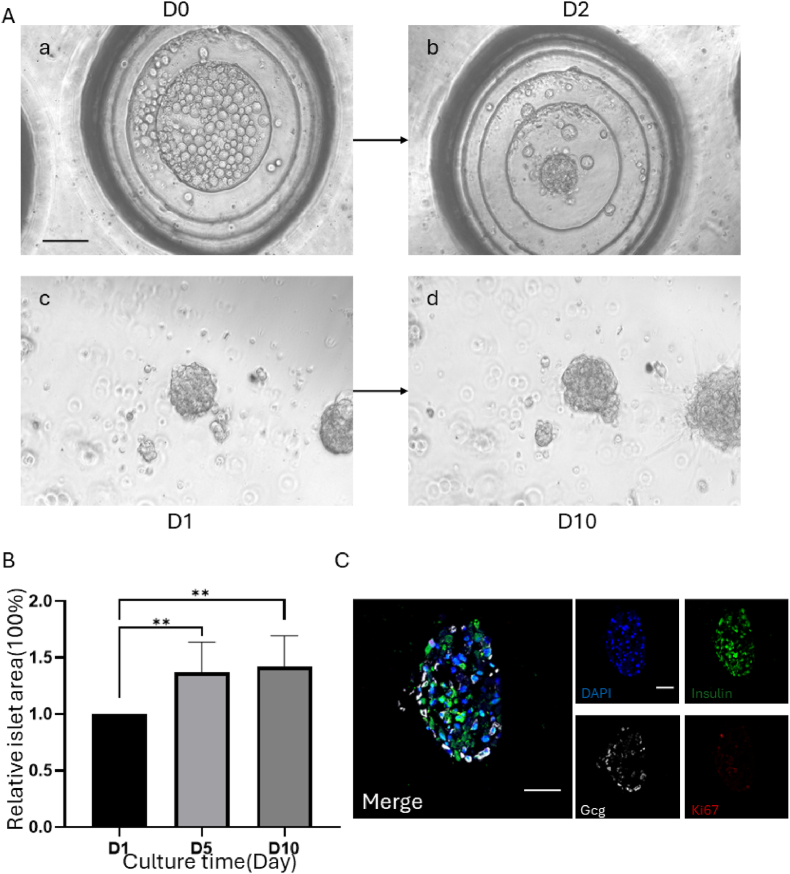
The passage of pancreatic islet clusters through microwells. A: Bright field diagram of pancreatic islet cells in a microplate aggregation assay after two days. Two days after CD, the pancreatic islets were transferred to the matrix gel and cultured until day 10; scale bar = 100 μm. B: The surface area of P1-generation pancreatic islet-like organs on days 1, 5, and 10, * *P < 0.01. C: P1-generation immunofluorescence staining of pancreatic islets, blue: DAPI, green: insulin, white: Gcg, red: Ki67, white: merge, scale bar = 50 μm.

Previous studies have indicated that single β cells are not able to perform their functions well, and the interactions between β cells and pancreatic islet cells, as well as the characteristic of being a complete small island, can help β cells better fulfill their functions. Therefore, we attempted to passage pancreatic islet-like organs through microplate aggregation. Figure ab shows that after 10 days of cultivation, NO4-containing organs were digested into single cells, at which point 5% matrix gel was added. After centrifugation of the microplate, the cells regrouped and formed clusters. After two days, the clusters did not separate, and the sizes and shapes of the formed clusters were consistent. The proteins were then transferred to the matrix gel. As shown in Fig. 5-A cd, after 10 days, the cell clusters exhibited corresponding expansion compared with the initial stage of clustering. Using ImageJ image analysis software, the areas of the tracked pancreatic islet cell clusters were calculated at different time points. As shown in Fig. 5-B, the cell clusters on day 10 exhibited a 1.5-fold increase compared with those on day 1. Then, immunofluorescence staining was performed for insulin, glucagon, and Ki67, as shown in Fig. 5-C. The P1 generation organoids could still maintain the secretion of insulin and glucagon, but Ki67 staining showed that the amplification ability was not as good as that of the P0 generation organoids (as shown in Fig. 3-A). Subsequently, we attempted to further passage the P2 generation, but due to insufficient (or very low) single-cell counts after digestion in the P1 generation, we were unable to proceed. In summary, studies on the passaging of pancreatic islet-like organs have shown that the islets can maintain transient expansion in the P0 and P1 generations in vitro and that the P1 generation still retains the characteristics of primary pancreatic islets.

### 2.6 Transplantation of pancreatic islets

**Fig. 6:**
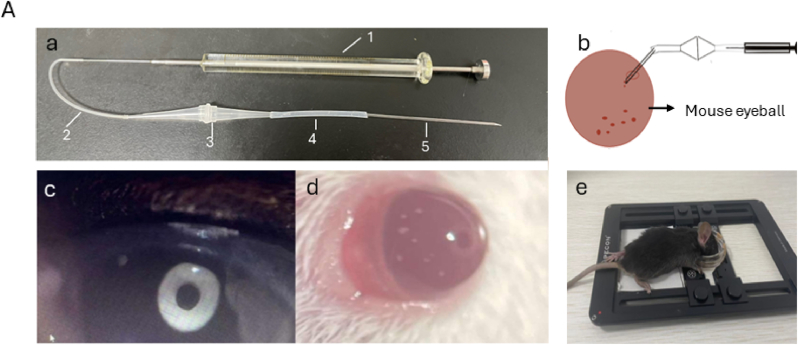
Schematic diagram of pancreatic islet transplantation into the anterior chamber of the eye. A, a: Tools required for mouse transplantation, 1: syringe, 2: rubber tube 1, 3: reservoir, 4: rubber tube 2, 5: glass tube b: schematic diagram of mouse eyeball transplantation. c,d: Bright-field diagram of islet transplantation into the anterior chamber of the mouse eye. e: Positioning of the mouse body during real-time live imaging.

In the mouse anterior chamber transplantation strategy reported in a 2013 study, more complex transplantation equipment was needed, and thus we simplified the process based on this study. Not only did the requirements for transplantation materials become more cost-effective, but the assembly of materials became simpler, and the experimental operation techniques became easier to perform. As shown in Fig. 6-A a, the device consists of a syringe, rubber tube 1, a 10 μl gun head, rubber tube 2, and a glass tube. As shown in Fig. 6-B, during transplantation, only the glass tube needed to be inserted into the mouse anterior chamber, and the assistant was required to quickly press the piston to inject the pancreatic islets into the mouse anterior chamber. After transplantation, the mice were treated with erythromycin ointment to prevent infection. Mouse transplantation can be observed under a stereomicroscope to obtain a clear field image of the transplanted pancreatic islets, as shown in Fig. 6-C. The transplanted pancreatic islets can be clearly seen in the anterior chamber of the eye. To observe the fluorescence map inside the mouse eyeball in real time, the following steps were followed to fix the mouse on the loading platform so that the eyeball was fully exposed at the bottom of the culture dish, as shown in Fig. 6-D.

## 3 Discussion

**Fig. 7:**
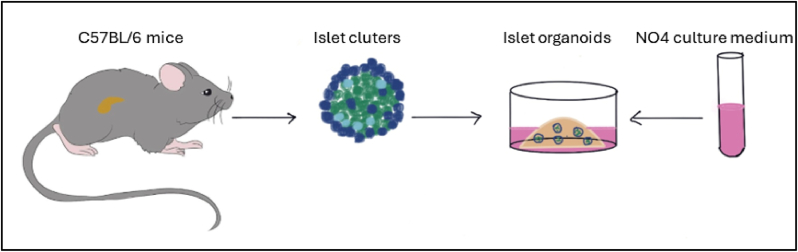
Diagram showing the expansion of primary pancreatic islet cells. After pancreatic islet clusters were isolated from C57BL/6 mice, the islets were cultured in NO4 medium to enable the in vitro expansion of primary pancreatic islet clusters from adult mice.

In 2020, one study reported a culture medium formula called PIEM, which could amplify pancreatic islet clusters isolated from pregnant and wild-type rats in vitro but failed to expand pancreatic islets from wild-type mice. Our study seamlessly filled this research gap. In this work, we developed a novel culture medium named NO4 for mouse islet expansion, as shown in Fig. 7. Compared with PIEM, NO4 exhibited significant differences in chemical composition. PIEM contains FGF10, Y-27632, gastrin, exendin 4, nicotinamide and NECA, whereas NO4 utilizes FGF2 and blebbistatin instead, and is further supplemented with Wnt3a, LDN193189, IGF-1, GABA and other key signaling modulators. Collectively, the optimized formulation enables NO4 to efficiently amplify pancreatic islet clusters isolated from wild-type mice and effectively induce insulin secretion.

The development process of the NO4 medium consisted of three stages, namely, the basal medium stage, the NO3 amplification medium stage, and the NO4 functional medium stage. When basal culture medium was used to culture isolated pancreatic islets, we found that the pancreatic islet clusters could not be expanded. Therefore, we consulted the literature and searched for factors that could promote the expansion of pancreatic islet clusters. The literature on cell proliferation and replication was screened for 7 small molecules, and it was ultimately found that the combination of WS6+GABA can better amplify pancreatic clusters, with a growth rate of 160%-180% over a 10-day culture cycle. To better expand pancreatic islet-like organs, the necessary small molecules were added to the culture medium to induce the differentiation of hPSCs into endocrine cells, and the resulting medium was named NO3 amplification medium. The expansion rate of the organoids in the NO3 group within 10 days was 200%-250%. Immunofluorescence showed that the organoids in the NO3 group expressed insulin and glucagon, and staining for these two markers indicated the presence of islets as well as β cells and α cells. This demonstrates that the organoids possessed the characteristics of pancreatic islet cells. Ki67 staining also confirmed that the organoids in the NO3 group exhibited strong expansion ability. However, qRT‒PCR revealed that the expression of Ins2 in the NO3 group of organoids was not as high as that in the overnight islets group. Therefore, to improve the insulin secretion ability of pancreatic organoids, we further developed the medium.

Through a literature review and high-throughput screening of small molecules, we identified the small molecule AS8351. When NO3 medium was added to pancreatic islet-like organs, we found that the growth rate of pancreatic islet clusters within 10 days was the same as that of the NO3 group, with an amplification rate of 200%-250%. The number of pancreatic islet clusters within 10 days also increased approximately two times compared with that on the first day, which is consistent with the changes in cell surface area mentioned earlier. Immunofluorescence showed that the NO4 group of organoids could express insulin and glucagon, and the expression of Ki67 showed strong amplification ability. According to quantitative RNA detection via qRT‒PCR, the NO4 group of organoids expressed more Ins2, and in the GSIS experiment, alternating stimulation of organoids with glucose demonstrated that the NO4 group of pancreatic islet organoids could secrete insulin. However, the actual stimulation magnitude and functional robustness still need to be further verified and quantified in our subsequent experiments.Therefore, we used NO4 medium as the final medium for the in vitro amplification of wild-type mouse pancreatic islet clusters and improved the amplification system. However, we did not perform in vivo experiments on mice, and we did not conduct functional transplantation experiments on humans, and therefore, further improvements are needed.

By recording the growth rate of pancreatic islet clusters, we found that the combination of the small molecules WS6 and GABA plays a very important role in the amplification stage. The literature reports that both GABA and WS6 can promote the growth of pancreatic islet cells. Consistent elevation in overall cell number was observed alongside the enlargement of morphological surface area, which collectively demonstrates that the expansion of islet organoids is driven by active cell proliferation, instead of passive cell spreading, cellular swelling or cell aggregation.Our experimental results verify these findings. The important role of the small molecule AS8351 in insulin secretion by pancreatic islet clusters was discovered through RNA quantification. Through RNA sequencing of NO3- and NO4-class organoids, it was found that NO3-class organoids expressed more genes related to amplification, while NO4-class organoids expressed more genes related to cell maturation. AS8351 is a KDM5B inhibitor. Pharmacological inhibition of KDM5B by AS8351 is critical for reconstructing the oocyte/2c-specific H3K4me3 domain, which facilitates the transition from pluripotency to multipotency [18]. Nevertheless, the potential association of the KDM5B–H3K4me3 regulatory axis with pancreatic and islet maturation is currently defined only as a preliminary hypothesis derived from bioinformatic evidence. Further in-depth experimental validations are therefore warranted to elucidate its exact role in regulating islet cell maturation.

Many methods for passaging organoids have been developed, such as mechanical isolation, coculture, and 3D printing. However, due to the weak regenerative ability and decreased passability of adult pancreatic islet cells, routine passaging methods are not suitable for adult pancreatic organoids. Moreover, since the pancreas itself is whole, forced digestion may damage the extracellular portion of the membrane or matrix proteins, which would result in impaired function. Therefore, individual cells cannot be expanded or passaged. Therefore, we regrouped the P0 generation cells that had been digested into single cells using microplate aggregation and then transferred them to matrix gel for further cultivation. Although this method can amplify the P0 and P1 generations of pancreatic islets and can amplify the P1 generation approximately 1.5 times within a week, immunofluorescence showed that the level of Ki67 was decreased in the P1 generation, and thus the amplification ability of P1 cells was limited. The impaired expansion potential of P1-generation islet organoids is likely caused by compromised cellular integrity and the disruption of the peripheral islet membrane structure, which provides essential structural protection. These membrane or matrix proteins may be key factors for in vitro tissue expansion. Moreover, the absence of endothelial cells may further restrict the proliferative activity of organoids. The insufficient cell abundance of the P1 generation, coupled with inevitable cell loss during the passaging operation, collectively leads to the failure of continuous subculture to the P2 generation.For further exploration, we will expand the sample size to repeat relevant experiments and optimize the current passaging protocols.Therefore, future analysis of the different expression patterns and functions of membrane proteins and signalling circuit differences between isolated islet clusters and islet cells may provide more clues for unravelling the secrets of the in vitro expansion of islets and provide guidance for a better strategy for in vitro islet cluster expansion.

Finally, to facilitate the subsequent in vivo transplantation of expanded islet organoids, we designed a simplified device for islet transplantation into the anterior chamber of the mouse eye, with reference to a previous protocol established in 2013. This device features simple assembly using only a small number of instruments, typically within 10 min, enabling rapid and convenient operative manipulation. Our results demonstrate that the expanded islet organoids can be successfully delivered and localized via this device, supporting the feasibility of in vivo application of our culture system.

Due to the limited expansion ability of adult tissues, most pancreatic islet-like organs currently originate from iPSCs or hPSCs and rarely originate directly from adult tissues, which results in the inability to achieve long-term expansion and passage like small intestine-like organs. Considering the convenience of adult tissue acquisition, engineering production and low immunogenicity, it would be easier to treat diseases related to pancreatic islets (such as diabetes and pancreatic cancer) with organoids derived from adult tissues. The newly established NO4 medium confers great application potential in disease modeling and translational research, which provides a robust and accessible in vitro platform for investigating metabolic and pancreatic disorders. This refined islet culture system permits high-throughput drug screening and preclinical validation of therapeutic candidates, thereby facilitating the functional assessment of small-molecule compounds in alleviating islet injury and restoring β-cell function. Furthermore, in combination with advanced genetic editing techniques, this culture platform is amenable to the construction of genetically tailored disease models for inherited pancreatic diseases. Collectively, the optimized NO4-based islet expansion system acts as a dependable and multifunctional experimental tool for mechanistic exploration, drug discovery, and translational investigation of pancreatic pathologies.Therefore, in the future, pancreatic islet-like organs should be more inclined to be derived from adult tissue sources.

In summary, our innovative research led to the development of a culture medium entirely composed of small molecules that can amplify pancreatic islet clusters isolated from wild-type mice. The pancreatic islet clusters can secrete insulin and are passaged in their complete form using a microporous aggregation method. In addition, we invented a more convenient and efficient mouse islet transplantation device in which islets were successfully transplanted into the anterior chamber of the eye of a mouse within 10 min.

## 4 Methods

### 4.1 Isolation and purification of pancreatic islets

1) Pancreatic perfusion: Six-month-old male wild-type mice were employed, as male mice possess more and larger pancreatic islets relative to female mice [32].The mice were anesthetized with pentobarbital sodium (or euthanized via spinal dislocation), the abdominal cavity was opened, and the common bile duct was identified and ligated under a dissecting microscope. Then, along the direction of the common bile duct, the hepatopancreatic ampulla of the duodenum was located. A 32G syringe needle was used to inject collagenase P into the pancreatic duct in the opposite direction from the hepatopancreatic ampulla until the entire pancreas (especially the tail of the pancreas, which may lose more pancreatic islets if not filled) was fully filled. Finally, the pancreas was manually removed.
2) Separation and purification of pancreatic islets: After the pancreas was removed, it was digested in a 37 °C water bath for 17 min, and digestion was terminated with 10% FBS + Hanks. Using a density gradient solution (Histopaque-1077), the sample was centrifuged at 2400 rpm for 22 min in a horizontal centrifuge to form a three-layer gradient solution. The solution containing the pancreatic islets in the middle layer was poured into a culture dish, and the pancreatic islets were manually selected under a microscope.
3) Cultivation of organoids: The selected islets were centrifuged, and the supernatant was removed until only the islet sediment remained. Matrix gel was used to resuspend the pancreatic islet precipitate in the EP tube. Then, 50-60 islets per well were placed in a 48-well plate and maintained in a 37 °C incubator at constant temperature for 10 min. After the matrix gel solidified, 200 μl of culture medium was added, and the culture was continued at 37 °C.

### 4.2 Statistics on the surface area and cell count of pancreatic islet clusters

1) Surface area statistics: The isolated pancreatic islets were buried in matrix gel. During organoid culture, a fixed time was selected, and 4-5 pancreatic islet clusters were randomly selected from each well of the plate. Then, the same 10X inverted microscope was used to obtain images, and the results were recorded and tracked. The experiment was repeated three times. The surface area of the obtained pancreatic islet organoids was calculated and analyzed using ImageJ software, and GraphPad Prism software was used to construct the growth field and growth rate curve of the pancreatic islet organoids.
2) Quantitative statistics: Pancreatic islets of similar size were selected under a microscope and randomly divided into two groups. Each group contained approximately 20 islets and received different treatments. After cultivation in overnight medium (RPMI 1640 + 10% FBS+1% P/S), the pancreatic islets from the overnight culture group were digested into single cells. The counting method involved a blood ball counting plate, and a counter was manually used to count the number of cells in each large square under a microscope (1 mm × 1 mm × 0.1 mm = 0.1 mm3). Independent sample t tests were used for comparison and will serve as a record of the initial number of cells in pancreatic islet-like organs. The other group of pancreatic islet-like organs was cultured in NO4 medium for 10 days and then digested into single cells using trypsin, after which the cells were counted in the same manner. This experiment was repeated 4 times; data were plotted using GraphPad Prism software and analyzed using SPSS software.

### 4.3 Immunofluorescence assay of pancreatic islet clusters

1) Embedding: First, the organoids were cleaned with cell recovery solution gel and PBS and then embedded in agarose (3%).
2) Dehydration, paraffin embedding, and sectioning: The embedded organoids were dehydrated in graded solutions of 75%, 85%, 90%, 95%, 100%, and 100% ethanol for 1 h, treated with xylene for 15 min or 2 times, treated with paraffin for 1 h or 2 times, and then embedded using an embedding machine. The embedded organoids could be sectioned the following day.
3) Antibody incubation: After sectioning, deparaffinization, and antigen retrieval, the sections were incubated with primary antibodies overnight at 4 °C, followed by incubation with the secondary antibody at room temperature for 1 h.

#### 4.3.1 qRT‒PCR detection of pancreatic islet clusters

1) RNA extraction: The RNA reagent extraction kit was used to extract RNA, and NanoDrop 2000c software was used to measure the concentration and purity of the RNA. RNA was extracted from the three groups of organoids, namely, the basal group, the NO3 group, and the NO4 group.
2) Reverse transcription of RNA extracted from the three groups of organoids into cDNA was performed using a 15 μl reaction mixture containing the following primers: 2x SYBR Green qPCR mix, 7.5 μl; primer mix, 1 μl; template mix, 1 μl, and ddH2O, 5.5 μl. The reaction conditions consisted of 40 cycles at 95 °C for 15 min, 95 °C for 15 s, and 60 °C for 30 s. In the experiment, 3 wells were tested for each gene in each group, and the qRT-PCR was repeated three times. The results were observed, compared and analyzed. After the reaction was completed, a detailed statistical analysis was conducted using GraphPad Prism software.

#### 4.3.2 Experimental measurement of insulin secretion under glucose stimulation

In this study, approximately 30 organoids were selected from the NO4 group. They were first washed three times in Krebs buffer and then preincubated in a low-sugar (2 mM) solution for 2 h to remove residual insulin. Subsequently, the cells were washed twice in Krebs buffer and incubated in a low-sugar solution for 30 min to collect supernatant samples. Subsequently, the cells were washed twice in Krebs buffer and incubated in a high-sugar (20 mM) solution for 30 min to collect the supernatant. This process was repeated twice. A mouse hypersensitive insulin/c-peptide ELISA kit was used to analyze insulin/c-peptide levels in the supernatant sample. Krebs buffer (12 mM HEPES, 121 mM NaCl, 5 mM KCl, 0.33 mM CaCl2, and 1.2 mM MgSO4; pH 7.4).

#### 4.3.3 RNA sequencing

The pancreatic islets of C57BL/6 wild-type mice were isolated and purified, and the mice were randomly divided into three groups: the NO3 group, the NO4-1 group, and the NO4-2 group. The pancreatic organoids were cultivated in their respective culture media for 10-14 days, after which RNA was collected from the three groups of pancreatic organoids. The collected RNA was transported to an external contract laboratory on dry ice for RNA sequencing, and complete genome maps and GSEA maps of the three groups of organoids were obtained.

#### 4.3.4 The passage of pancreatic islet clusters

1) Mold fabrication: First, the 24-well plate mold was placed in F127 overnight, the overnight mold was allowed to dry, and then 100 μl of curing agent was added to the 24-well plate; then, 1 ml of polydimethylsiloxane (PDMS) was added to the plate (the front part of the gun head was cut off before adding PDMS to avoid blockage). After the two reagents were added, a 200 μl gun head was used to remove small bubbles, after which the plate was covered and freeze-dried. This process resulted in the production of a microporous aggregation plate.
2) Pancreatic single-cell clusters: First, the well plates were prepared and thoroughly rinsed with PBS until no bubbles were observed. Cell recovery resolution gel was used to digest pancreatic islet-like organs into single cells using trypsin. Then, 100-200 μl of cell suspension/5% matrix gel was added and gently spread evenly on the micropores. The suspensions were centrifuged at 300-400 × g for 3-5 min. After centrifugation, the cells were observed under a microscope, and 500 μl of culture medium was gently added along the sidewall. The cells were incubated in a 37 °C incubator for two days. Images were captured and recorded.
3) Transfer of pancreatic islet cell clusters: The pancreatic islet cell clusters that had accumulated in the microplate for two days were transferred by gently blowing them with a gun head. After centrifugation and precipitation, the cells were resuspended in matrix gel, culture medium was added, and the cells were cultivated for approximately 10 days. Images were obtained and recorded.

#### 4.3.5 Islet eyeball transplantation

First, the islet transplantation device was assembled as shown in Fig. 6A–a; the culture dish was rotated in a small circle, and the islets were placed in the center of the dish. By adjusting the piston, the pancreatic islets were sucked from the gun head into the "reservoir" composed of the gun head, and then rubber tube 2 was connected to the gun head. By manually popping a rubber tube, the pancreatic islets collected in a single state at the outlet of the glass tube. If gas was present in the front section of the outlet, it was expelled. Then, the mice were anesthetized by intraperitoneal injection of anesthetic drugs. The assistant exposed the mouse eyeballs under a microscope and then used an insulin needle to poke a window in the cornea. The glass tube entered the anterior chamber along the window, and the assistant was then asked to quickly press the piston to inject the pancreatic islets into the mouse's anterior chamber. This procedure was stopped before or after all bubbles in front of the pancreas were flushed out of the glass tube. If the assistant was unsure, the procedure was stopped when the pancreatic islets entered the glass tube because the remaining bubbles in front of the islets helped prevent them from flowing back out of the anterior chamber. After transplantation, the mice were treated with erythromycin ointment to prevent infection. In mouse transplantation, bright field images of transplanted islets can be obtained under a stereomicroscope.

## Author Contribution

Lingxian Zhang and Kairui Liu participated in the conceptualization, methodology, investigation and writing-original draft preparation. Xiaoxiao Zhang and Yubing Cui participated in the methodology, investigation and proofreading. Xiaolei Hu and Hao Hu participated in the supervision, resources, writing reviewing and editing.

## Ethical statement

Handling of all experimental mice used in this study was approved by the Animal Ethics Committee of Bengbu Medical College (Approval No. 2021-259) and performed in strict accordance with the ARRIVE guidelines.

## Funding

This study was supported by the 10.13039/501100003995Natural Science Foundation of Anhui Province (Grant No. 2208085MH216), the Major Natural Science and Technology Project of Bengbu Medical College (Grant No. 2020byfy004).

## Declaration of competing interest

The authors declare that they have no known competing financial interests or personal relationships that could have appeared to influence the work reported in this paper.

## Data Availability

The materials described in the manuscript, including all relevant raw data, will be freely available to any researcher wishing to use them for non-commercial purposes, without breaching participant confidentiality.
