# Supplementary material for: Small molecules promote the in vitro expansion and passaging of adult mouse pancreatic islets
Source: Biochem Biophys Rep. 2026 May 20;46:102622. doi: 10.1016/j.bbrep.2026.102622 (PMC13214305; doi:10.1016/j.bbrep.2026.102622)
Supplement: Multimedia component 1 [file mmc1.docx]

**Supplementary Table 1**

| Ingredient | Volume or Concentration |
| --- | --- |
| Advanced DMEM F12 | 50 ml |
| GlutaMax | 500 µl |
| B27 | 1 ml |
| N-Ace(0.5 M) | 100 µl |
| P/S | 200 µl |
| R-Spondin-1 | 500 ng/ml |
| EGF | 50 ng/ml |
| FGF2 | 10 ng/ml |
| A83-01 | 50 nM |

**Note:** Table 1 shows the required components and concentrations for NO1 cultivation medium.

**Supplementary Table 2**

| Ingredient | Volume or Concentration |
| --- | --- |
| Advanced DMEM F12 | 50 ml |
| GlutaMax | 500 µl |
| B27 | 1 ml |
| N-Ace(0.5 M) | 100 µl |
| P/S | 200 µl |
| R-Spondin-1 | 500 ng/ml |
| EGF | 50 ng/ml |
| FGF2 | 10 ng/ml |
| A83-01 | 50 nM |
| WS6 | 1 µM |
| GABA | 100 µM |

**Note:** Table 2 shows the required components and concentrations for NO2 cultivation medium.


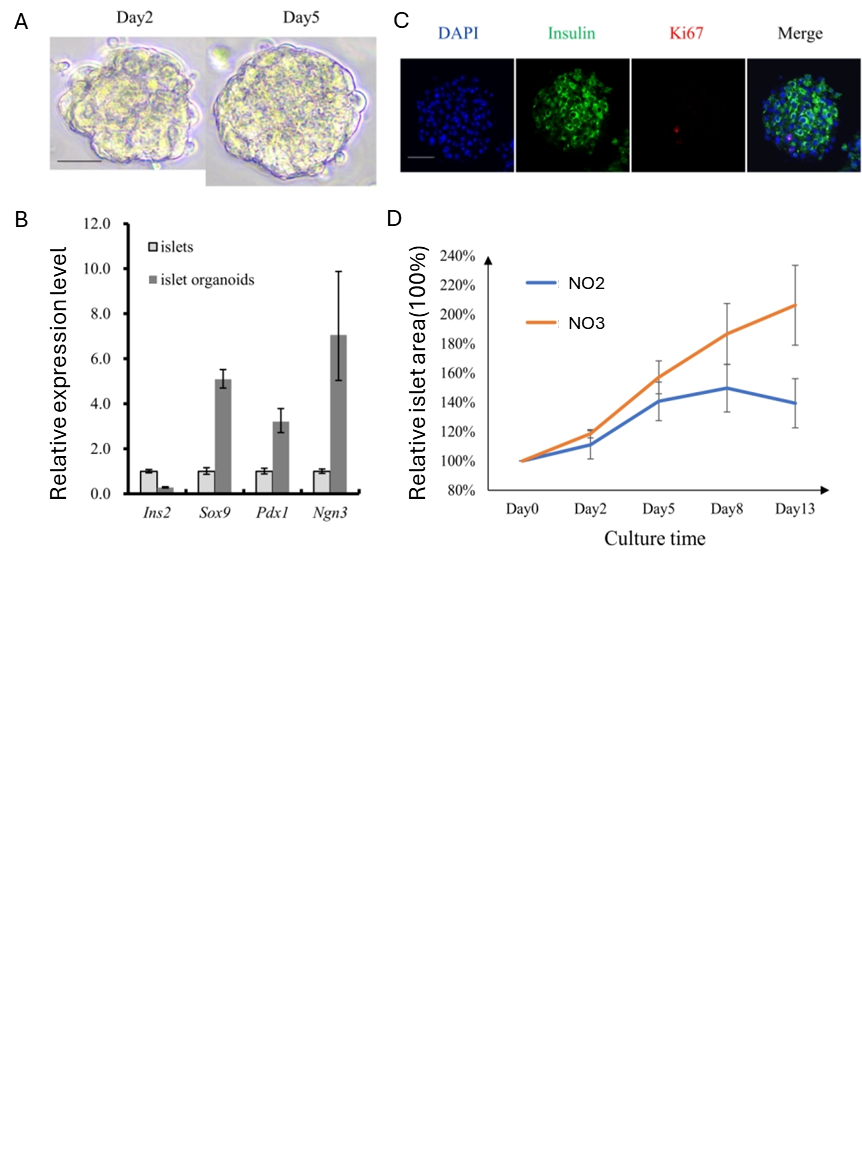
**Figure S1 In vitro amplification of pancreatic islet-like organs** A: Bright field images of pancreatic islet-like organs on the 2nd and 5th days under NO1 medium conditions were obtained using an inverted microscope at 10X magnification; scale bar=100 µm; B: qRT‒PCR experiments were conducted on overnight islets and islet-like organs cultured in NO1 medium for 2 weeks, with 4 wells in each group. The relative expression levels were calculated using the 2- △ △ Ct method based on the Ct value; C: Immunofluorescence staining of pancreatic islet-like organs under NO1 culture conditions, blue: DAPI, green: insulin, red: Ki67, scale bar=100 µm; D: Growth rate curves of pancreatic islet-like organoids in NO2 and NO3 media.
