# Supplementary material for: Small molecules promote the in vitro expansion and passaging of adult mouse pancreatic islets
Source: Biochem Biophys Rep. 2026 May 20;46:102622. doi: 10.1016/j.bbrep.2026.102622 (PMC13214305; doi:10.1016/j.bbrep.2026.102622)
Supplement: Multimedia component 2 [file mmc2.docx]

**Supplementary Table 2**

| Ingredient | Volume or Concentration |
| --- | --- |
| Advanced DMEM F12 | 50 ml |
| GlutaMax | 500 µl |
| B27 | 1 ml |
| N-Ace(0.5 M) | 100 µl |
| P/S | 200 µl |
| R-Spondin-1 | 500 ng/ml |
| EGF | 50 ng/ml |
| FGF2 | 10 ng/ml |
| A83-01 | 50 nM |
| WS6 | 1 µM |
| GABA | 100 µM |

**Note:** Supplementary Table 2 shows the required components and concentrations for NO2 cultivation medium.
